# Helios + Regulatory T cell frequencies are correlated with control of viral replication and recovery of absolute CD4 T cells counts in early HIV-1 infection

**DOI:** 10.1186/s12865-017-0235-7

**Published:** 2017-12-16

**Authors:** Raquel Matavele Chissumba, Eduardo Namalango, Vânia Maphossa, Ivalda Macicame, Nilesh Bhatt, Christina Polyak, Merlin Robb, Nelson Michael, Ilesh Jani, Luc Kestens

**Affiliations:** 1 Instituto Nacional de Saúde, Maputo, Mozambique; 2 Institute of Tropical Medicine, Department of Biomedical Sciences, Antwerp, Belgium; 3 Department of Biomedical Sciences, University of Antwerp, Antwerp, Belgium; 4 Military HIV Research Program, Walter Reed Army Institute of Research, MD, USA

**Keywords:** Tregs, HIV early infection, Helios

## Abstract

**Background:**

The acute phase of HIV infection is characterized by massive depletion of CD4 T cells, high viral plasma levels and pronounced systemic immune activation. Regulatory T cells (Tregs) have the potential to control systemic immune activation but also to suppress antigen specific T and B cell response. The co-expression of FoxP3 and Helios transcription factors, has been described for identification of highly suppressive Tregs. The aim of this study was to characterize the phenotype of classic Tregs during early HIV infection, and to assess the correlations between the frequencies and phenotype of Tregs with the plasma viral load, CD4 counts, immune activation and the frequency of antibodies reactive to HIV-1 proteins, measured by an immunochromatographic test.

**Results:**

The relative frequency of classic Tregs cells in peripheral blood correlated positively with HIV viral load and immune activation of CD8 T cells, and inversely with absolute CD4 counts and development of anti-HIV antibodies in subjects with early HIV infection. However, the expression of Helios in classic Tregs was inversely correlated with viral replication and immune activation, and positively with recovery of CD4 T cell counts and appearance of antibodies reactive to HIV-1 proteins.

**Conclusion:**

These results raise the hypothesis that classic Tregs are inefficient at controlling systemic immune activation in subjects with early HIV infection and may be associated with delayed production of antibodies against HIV proteins, delaying the control of viral replication. Conversely, Helios expressing Tregs might contribute to control of viral replication by mechanisms involving the limitation of systemic immune activation.

**Electronic supplementary material:**

The online version of this article (10.1186/s12865-017-0235-7) contains supplementary material, which is available to authorized users.

## Background

Regulatory T cells (Tregs) are a subset of CD4 T cells with potential to suppress the immune response, preventing excessive immune activation. Immune suppression by Tregs is mediated by soluble factors or by mechanisms requiring cell-to-cell contact [1]. To date several cells with regulatory properties have been described, but the classic Tregs are the most extensively studied.

Classic Tregs have been characterized by high expression of the activation marker CD25, presence of the transcription factor Foxp3 or expression of low levels of the alpha chain of Interleukin-7 (IL-7) receptor, CD127 [2]. In addition, classic Tregs can be classified as natural or induced Tregs [3, 4]. Natural Tregs emerge from the thymus with regulatory properties while induced Tregs are generated in the periphery following T cell activation [5, 6]. The co-expression of the transcription factors Helios and FoxP3 identifies highly suppressive Tregs, initially identified as natural Tregs [7, 8].

The role of Tregs preventing the development of exacerbated reactions during the course of an immune response has been highlighted in autoimmune diseases [9], transplantation [10], tumors [11] and infectious diseases [7], including HIV infection [12]. In HIV infection Tregs can be beneficial by controlling chronic immune activation, but also unfavorable or even harmful by preventing development of humoral and cellular anti-HIV immune responses [12–14]. A relative increase of classic Tregs in peripheral blood has been observed in treatment-naive patients with chronic progressive HIV infection, compared with healthy uninfected individuals [15, 16]. In addition, it has been suggested that virologic control induces a decrease in classic Tregs [17]. Little information, however, is available on the role of classic Tregs during early HIV infection, particularly in subtype C infections, the most prevalent HIV subtype in Sub-Saharan Africa and globally [18].

The early phase of HIV infection is critical as it determines the fate of the progressive infection [19–21] and the extent of virus transmission at population level [22, 23]. The early phase starts with an exponential rise in HIV viremia, reaching a peak when acquired cellular and humoral immune responses against viral proteins emerge [23].

The aim of this study was to characterize the phenotype of Tregs in individuals with early HIV infection, and to assess how staging of the infection, based on plasma viral load, immune activation, absolute CD4 T cell counts and the frequency of antibodies reactive to HIV-1 proteins, correlates with the relative abundance and phenotype of Tregs. We report opposite correlations between total classic Tregs and those expressing Helios, during early HIV-infection, with the plasma viral load, T cell activation, development of anti-HIV antibodies and absolute counts of CD4 T cells.

## Methods

### Study participants

A total of 37 individuals, 14 with early HIV infection (< than 3 months), 8 with HIV chronic infection (> 9 months) and 15 uninfected controls were enrolled in this study. All subjects were part of a large cohort study (RV363) established for assessment of HIV incidence and associated risk factors, and willingness to participate in future HIV vaccine trials. The HIV incidence study enrolled participants in Maputo, Mozambique, at Centro de Investigação e Treino em Saúde da Polana Caniço (CISPOC) between December 2013 and November 2014. A total of 500 uninfected individuals between 18 and 35 years, at high risk for HIV infection, were enrolled for assessment of HIV incidence every three months, over a period of two years. A smaller group of HIV-infected individuals, as part of the chronic infection group, were also included in the study, as a community masking purposes. Pregnant women were excluded from the study. Peripheral blood samples were collected from HIV early infected participants, those whom the rapid test shift from negative to positive in a period of 3 months (median) and masking group individuals for assessment of virologic and immunologic parameters. For the Tregs sub-study, peripheral blood mononuclear cells (PBMC) were available for 14 out of 19 individuals who seroconverted during the HIV incidence study. In addition, PBMC from 3 seroconverted individuals and 5 chronically infected individuals, were evaluated 9 months after the first PBMC collection and at least 2 years after infection, respectively. Randomly selected individuals who did not seroconvert and were negative for HIV, hepatitis B virus (HBV), hepatitis C virus (HCV) and syphilis were considered as control subjects (uninfected). Immunophenotyping of PBMCs was done on 22 HIV infected individuals and 15 uninfected controls.

### HIV diagnosis, CD4 counts and viral load testing

**Fig. 1:**
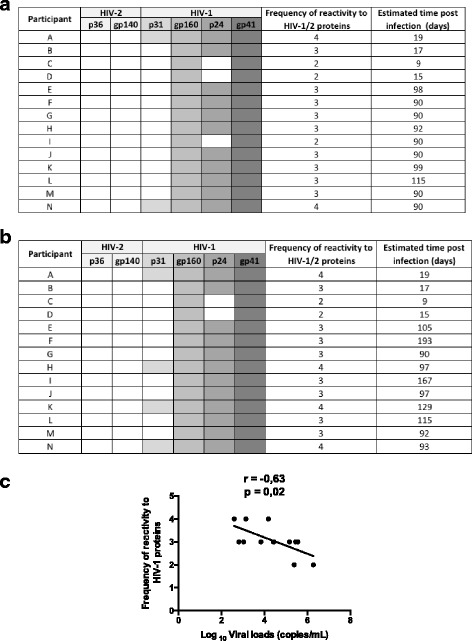
Frequency of antibodies reactive to HIV-1 proteins in plasma from HIV early infected individuals. Serum samples from HIV infected individuals with early infection were collected for assessment of reactivity to HIV-1 proteins using the Geenius HIV-1/2 Confirmatory System. The reactivity of serum samples to HIV-1 proteins p31, gp160, p24 and gp 41 was recorded at seroconversion visit (**a**) and at time of PBMC collection (**b**). For each individual, the frequency of reactive bands on Geenius test, is indicated. **c** Correlation between the frequency of reactive bands and viral loads at PBMC collection visit

The HIV-1 diagnosis was performed on fresh venous whole blood samples, following the Mozambican national algorithm for HIV testing, which consists of two sequential rapid immunochromatographic tests for detection of anti-HIV-1/2 antibodies. Screening was first performed using the *Alere Determine™ HIV-1/2* (Alere Medical Co., Japan) rapid test. Non-reactive specimens were classified as negative. Reactive specimens were confirmed by a second rapid test, the *Uni-Gold™ HIV* (Trinity Biotech PLC, Ireland). Indeterminate results, reactive for Determine but non-reactive for UniGold, were resolved by a fourth-generation ELISA, *Genscreen Ultra HIV Ag-Ab* (BioRad, France). The antibody reactivity pattern for HIV-1 (p31, gp160, p24 and gp41) proteins was analyzed in all seroconverted individuals, using the *Geenius HIV 1/2 Confirmatory Assay* (BioRad, France) at seroconversion visit and at time of PBMC collections (Fig. 1a and b). CD4 counts were performed on whole blood cells as previously described [24]. Acquisition was performed on the four-color flow cytometer FACS CALIBUR (BD, USA). Plasma HIV-1 viral loads were determined using the *COBAS AmpliPrep/COBAS TaqMan HIV-1 Test, v2* (Roche, USA).

### Peripheral blood mononuclear cells immunophenotyping

Peripheral blood mononuclear cells (PBMC) were isolated from freshly obtained heparin anti-coagulated blood, using Ficoll-Paque Plus (GE Healthcare, Sweden) and Leucosep tubes (Greiner Bio-One, German), and stored in liquid nitrogen, in a freezing media (10% dimethyl sulfoxide (DMSO) + 90% fetal calf serum (FCS)). After thawing in a water bath at 37°C, PBMC were washed twice in complete RPMI medium supplemented with 20% of FCS followed by 10% of FCS (R20 and R10, respectively) and viable cells were counted using the Nucleocounter NC-100 (Chemometec, Denmark). Prior to cell staining, PBMC were allowed to recover overnight in R10 medium at 37 °C in an atmosphere of 7.5% CO_2_. Subsequently, PBMC were counted and washed in phosphate buffered saline (PBS). Cell viability was assessed by adding 50 μl of the viability dye (fixable viability stain (FVS) 510 (BD, USA)) to one million PBMC. Finally, cells were washed again and stained for cell surface markers. Three eight-color panels were prepared per subject, each containing 500,000 cells. One panel was used to stain cell surface markers only and two other panels were used for combined staining of surface markers and transcription factors, FoxP3 and Helios, all following the instructions from the manufacturer. The FoxP3 buffer set from BD was used to fix and permeabilize PBMC for FoxP3 and Helios staining following manufacturer recommendations.

The following eight-color panels were used: (1) CD3^FITC^/ CD45RA^PerCP^/ HLADR^PE-CY7^/ CD38^APC^/ CD8^APC-H7^/ CD4^V450^/ FVS510 for assessment of levels of immune activation of CD4 and CD8 T cells; (2) CD195^FITC^/ FoxP3^PE^/ CD49d^PerCP^/ CD25^PE-CY7^/ B7^APC^/ CD3^APC-H7^/ CD4^V450^/ FVS510, and (3) CD31^FITC^/ FoxP3^PE^/ CD45RA^PerCP^/ CD25^PE-CY7^/ Helios^APC^/ CD3^APC-H7^/ CD4^V450^/ FVS510 were used for co-expression analysis of lineage or activation markers and expression of HIV-1 co-receptors on classic Tregs. After staining, cells were fixed with 1X BD CellFix (BD, USA) and analyzed within 24 h. The description of each monoclonal antibody is summarized in Additional file 1. Extensive titration experiments were performed on monoclonal antibodies prior to testing of participant samples.

A minimum of 100,000 events were acquired on FACSCanto II (Becton Dickinson (BD), USA) with Diva software version 8 (Becton Dickinson, USA). The BD Cytometer Setup & Tracking (CST) beads and BD Comp beads were used to ensure consistency of results over time and for compensation, respectively. The post-acquisition analyses, including compensation, were performed using the FlowJo software, version 10 (FlowJo LLC, USA). The fluorescence minus one (FMO) controls were used for definition of positivity applied to markers with continuous or dim levels of expression during post acquisition analysis. Representative plots, showing the gating strategy used for definition of T cells, including the classic Tregs and their phenotypes, are shown in Additional file 2 and Additional file 3.

### Statistical analysis

Statistical analyses were performed using the software *GraphPad Prism* version 6.0 h (San Diego, CA). The *Mann-Whitney* test was used to test the heterogeneity among different groups. The analysis of correlations between two variables was performed by the *Spearman Rank correlation*. Difference or correlation with *p*-values less than 0.05 were considered statistically significant.

## Results

Characterization of subjects with early HIV infection. An overview of characteristics of the study participants is shown in Table 1. In total, 14 early infected individuals were included in this sub-study, of whom seven were female. The median age was 21 years and none of the participants had received any HIV antiretroviral treatment at time of PBMC collection. The time between the last HIV negative test and first positive rapid test were 90 days (median) while the PBMC collections were performed 95 days (median) after infection. The median viral load at time of PBMC collection was 4.42 Log10 copies/mL. As expected, the viral load was inversely correlated with the frequency of antibody reactive bands to HIV-1 proteins (*r* = −0.63; *p* = 0.02) as shown in Fig. 1c. For some participants, the number of reactive bands increased from between the seroconversion visit and the time of PBMC collection (Fig. 1a and b).

**Table 1:** Characteristics of study groups

	Early (HIV^+^)	Chronic (HIV^+^)	Control (HIV^−^)
N	14	8	15
Median age (IQR) (years)	21 (20–24)	23 (21–33)	20 (19–24)
Sex (F/M)	7/7	6/2	10/5
Median #CD4 (IQR) (cells/μL)	628 (396–771)	626 (492–749)	902
Median log_10_ viral load (IQR) (copies/mL)	4.43 (3.11–5.40)	4.61 (2.32–4.93)	N/A
Median time before the last negative HIV rapid test and the first positive result (days)	93	>270	N/A
Participants with viral load detected or >20 copies/mL (N)	14	4	N/A
Participants on ART (N)	0	7	N/A

*IQR* interquartile range, *M/F* male/female, *N/A* not applicable, *ART* Antiretroviral therapy

### CD8 T cell activation correlates with the frequency of reactive bands to HIV-1 proteins in peripheral blood of patients with early infection

It is well known that HIV infection and disease progression is associated with systemic immune activation. T-cell activation starts at the acute phase of HIV infection and persists during the chronic phase, even after initiation of highly effective antiretroviral therapy [25]. Both CCR5 and CCR3 participate in T cell activation and migration during the inflammatory process, and are expressed predominantly on memory Th1 cells [26].

**Fig. 2:**
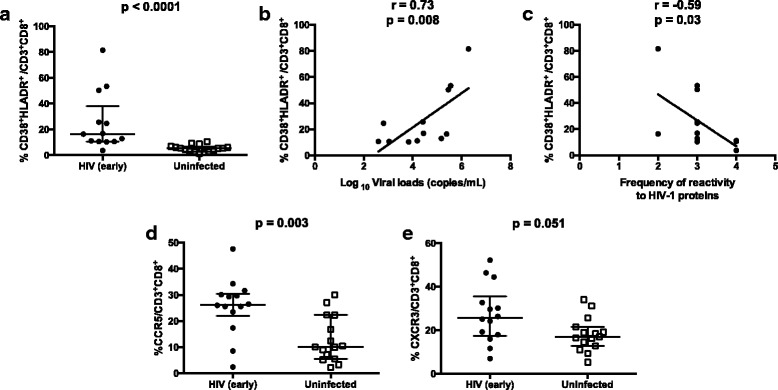
Proportions of CD8 T cells co-expressing the activation markers and chemokine receptors. PBMC from 15 HIV uninfected individuals and 14 individuals early infected by HIV-1, were isolated and manipulated as described in methods section. These PBMC, were stained with monoclonal antibodies and analyzed by polychromatic flow cytometry. Viral loads and frequency of antibodies reactive to HIV-1 proteins was determined using plasma samples and serum, respectively, as described on methods section. **a** Median and interquartile range of proportions of CD8 T cells co-expressing the activation markers CD38 and HLA-DR between uninfected and HIV early infected groups. Each point represents an individual. Unpaired Mann-Whitney test *p*-values, comparing medians from HIV uninfected group and individuals with HIV early infection, are indicated on each figure. **b**-**c** Correlation analysis between the immune activation of CD8 T cells with the viral loads and frequency of antibodies reactive to HIV-1 proteins in HIV-1 early infected subjects. Spearman correlation r and *p*-values are indicated on each figure. **d**-**e** Median and IQR of proportions of CD8 T cells expressing the chemokine receptors, CCR5 and CXCR4. Each point represents an individual. Unpaired Mann-Whitney test p-values, comparing medians from HIV uninfected group and individuals with HIV early infection, are indicated on each figure

CD8 T cell activation was high in HIV early-infected group as compared to the HIV negative control group (*p* < 0.0001), and correlated positively with viral load (*r* = 0.73; *p* = 0.008) and inversely with the frequency of antibodies reactive to two or more HIV-1 proteins, namely p31, gp160, p24 and, gp4 (*r* = −0.59; *p* = 0.03) (Fig. 2a–c). We found an increased proportion of CD8 T cell (CD3^+^CD8^+^) expressing CXCR3 (*p* = 0.05) and CCR5 (*p* = 0.002), as compared to controls (Fig. 2d and e). However, the proportion of CD8 T cells expressing CCR5 and CXCR3 was not associated with either viral load (*r* = 0.01; *p* = 0.9 and *r* = 0.17; *p* = 0.57, respectively) or frequency of antibody responses against HIV-1 proteins (*r* = 0.20; *p* = 0.48 and *r* = −0.18; *p* = 0.71, respectively).

The activation profile of CD4 T cell (CD3^+^CD4^+^), measured by analyzing the co-expression of HLA-DR and CD38, was significantly higher in the HIV early infected group as compared to controls (*p* = 0.02). The proportions of both CD4^+^CD38^+^HLADR^+^ and CD4^+^HLADR^High^ tended to correlate inversely with the frequency reactive bands of HIV proteins (*r* = −0.45; *p* = 0.12 and *r* = −0.66; *p* = 0.01, respectively) and with the absolute CD4 T cell counts (*r* = −0.51; *p* = 0.07 and r = −0.4; *p* = 0.17, respectively). However, no significant correlation was found between the CD4 T cell activation and viral load (=0.34; *p* = 0.26). We also found a positive correlation between the percentage of recent CD4 thymic emigrants, defined as CD3^+^CD4^+^CD31^+^CD45RA^+^, and the frequency of reactivity to HIV-1 proteins (*r* = 0.6; *p* = 0.04), but percentages did not differ between HIV early infected patients and controls (*p* = 0.45).

With regard the expression of chemokine receptors on CD4 T cells, we did not find significant differences of CD4^+^CCR5^+^ (*p* = 0.24) and CD4^+^CXCR3^+^ T cells (*p* = 0.97) expression levels between the two groups. In addition, the proportion of CD4^+^CCR5^+^ (*p* = 0.24) and CD4^+^CXCR3^+^ T cells did not correlate significantly with viral load (*r* = −0.16; *p* = 0.59 and *r* = −0.16; p = 0.59, respectively) or with the frequency of antibody response against HIV proteins (*r* = 0.15; *p* = 0.60 and r = 0.15; *p* = 0.60, respectively).

### Proportions of classic CD4^+^CD25^High^FoxP3^+^ Treg cells correlates with viral load, reactivity to HIV-1 proteins, and CD4 counts in HIV patients with early infection

**Fig. 3:**
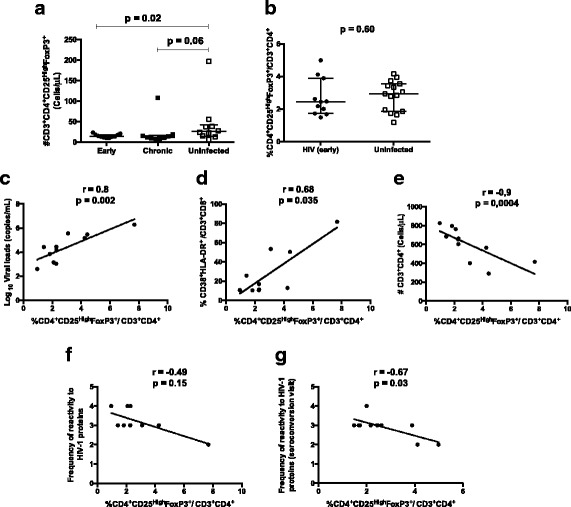
Frequencies of Tregs and correlations with virologic and immunologic indicators. PBMC from HIV uninfected controls (15), HIV recently (11) and chronically (eight) infected patients, were isolated and manipulated as described in methods section. These PBMC were stained with monoclonal antibodies and analyzed by polychromatic flow cytometry. Viral loads and frequency of antibodies reactive to HIV-1 proteins were determined using plasma and serum samples, respectively, as described on methods section. **a-b** Median and interquartile range for proportions and absolute counts of classic Tregs, CD4^+^CD25^High^FoxP3, in study groups. Each point represents an individual. Unpaired Mann-Whitney test *p*-values comparing medians from controls and HIV infected groups are indicated on each figure. **c-g** Correlations between the proportions of classic CD4^+^CD25^High^FoxP3^+^ Tregs with the viral loads, immune activation, absolute CD4 T cell counts and frequency of antibodies reactive to HIV-1 proteins at time of PBMC collections and at seroconversion visit. Each point corresponds to one individual. Spearman correlation r and *p*-values are indicated on each figure

The absolute counts and percentages of classic Tregs was analyzed. We observed a decrease of absolute numbers of classic CD4^+^CD25^High^FoxP3^+^ Tregs in HIV early infected patients compared with control group (*p* = 0.02) but not with those with chronic infection. No difference was observed in proportion when compared with control group (p = 0.60) (Fig. 3a and b). However, the proportion of classic Tregs, in the HIV early infected group correlated positively with viral load (*r* = 0.8; *p* = 0.002), the proportion of activated CD8^+^CD38^+^HLADR^+^ (*r* = 0.68; *p* = 0.03) cells, and absolute numbers of CD4 T cells (*r* = −0.9; *p* = 0.0004) (Fig. 3c-e). Despite the absence of significant correlation between the frequency of Tregs and the frequency of bands reactive to HIV proteins (*r* = −0.53; *p* = 0.1), at time of PBMC collection, we found a significant correlation with the number of bands at seroconversion visit (*r* = −0.67; *p* = 0.03) (Fig. 3f and g).

The absolute decrease of classic Tregs did not correlate with viral load (*r* = 0.21; *p* = 0.53), frequency of antibodies reactive to HIV-1 proteins (*r* = −0.26; *p* = 0.43) nor total number of CD4 T cells (*r* = −0.17; *p* = 0.61).

### Increased expression of CCR5 and CXCR3 on classic CD4^+^CD25^High^FoxP3^+^ Tregs in HIV patients with a recent infection

Chemokine receptor expression on Tregs can predict their function and their potential to accumulate in non-lymphoid tissues [27]. It has been shown that CXCR3^+^ and CCR5^+^ Tregs are recruited to sites with ongoing Th1 inflammation [28, 29]. Furthermore, increased expression levels of CCR5 and integrin β7 are associated with higher susceptibility to HIV infection and poor immunological response [30–32].

**Fig. 4:**
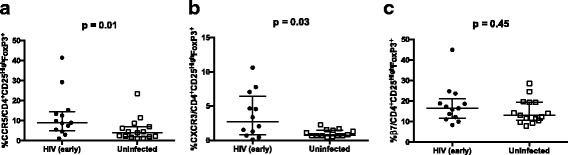
Expression of chemokine receptors CCR5 and CXCR3, and β7 integrin in classic Tregs. PBMC from 15 HIV uninfected individuals and 11 HIV-1 early infected individuals, were isolated and manipulated as described in methods section. These PBMC were stained with the following combination of monoclonal antibodies: CD195^FITC^/ FoxP3^PE^/ CD49d^PerCP^/ CD25^PE-CY7^/ β7^APC^/ CD3^APC-H7^/ CD4^V450^/ FVS510. Data acquisition was performed on a flow cytometer (FACS CANTO II). Viral loads and frequency antibodies reactive to HIV-1 proteins was determined using plasma samples as described on methods section. **a-c** Median and interquartile range for proportions of CCR5, CXCR3 and β7 expression on classic CD4^+^CD25^High^FoxP3^+^ Tregs, in study groups. Each point represents an individual. Unpaired Mann-Whitney test p-values comparing medians from HIV uninfected controls and HIV infected group are indicated on each figure. Each point corresponds to one individual. p-values are indicated on each figure

We observed an increased proportion of CCR5 (*p* = 0.01) and CXCR3 (p = 0.03) on classic Tregs in patients with a recent HIV infection as compared to controls (Fig. 4). However, the increased expression of CCR5 and CXCR3 on classic Tregs did not correlate with viral load, nor with reactivity to HIV-1 proteins or with T cell activation.

The proportion and absolute number of classic CD4^+^CD25^High^FoxP3^+^ Tregs expressing β7 integrin did not differ between HIV patients and controls (*p* = 0.45 and *p* = 0.22 respectively). Their expression did not correlate with viral load, nor with frequency of antibodies reactive to HIV-1 proteins, T cell activation or with absolute numbers of CD4 T cells.

### The proportion of Helios expressing Tregs correlates with HIV-1 viral loads and CD4 T cell absolute counts

**Fig. 5:**
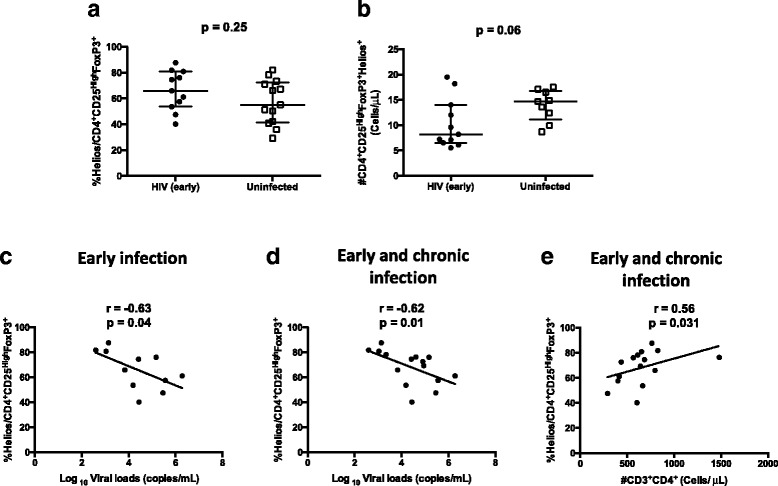
Frequencies of Helios expressing Tregs and correlation with virologic and immunologic indicators. PBMC from HIV uninfected controls (15), HIV recently (11) and chronically (eight) infected patients, were isolated and manipulated as described in methods section. These PBMC were stained with the following combination of monoclonal antibodies: CD31^FITC^/ FoxP3^PE^/ CD45RA^PerCP^/ CD25^PE-CY7^/ Helios^APC^/ CD3^APC-H7^/ CD4^V450^/ FVS510. Data acquisition was performed on a flow cytometer (FACS CANTO II). Viral loads and frequency of antibodies reactive to HIV-1 proteins was determined using plasma samples as described on methods section. Absolute frequency of CD4 T cells was determined using a four-color flow cytometer (FACS Calibur), and the TruCount method, as described previously described [24]. **a**-**b** Median and interquartile range for proportions and absolute counts of CD4^+^CD25^High^FoxP3^+^Helios^+^ Tregs in study groups. Each point represents an individual. Unpaired Mann-Whitney test p-values comparing medians from controls and HIV infected group are indicated on each figure. **c** Correlation between the frequency of Helios expressing Tregs in early infected subjects with the viral loads. **d**-**e** Correlation between the frequency of Helios expressing Tregs in early and chronically infected subjects with the viral loads and absolute CD4 T cell counts. Each point corresponds to one individual. Spearman correlation r and p-values are indicated on each figure

It has been shown that co-expression of FoxP3 and Helios identifies Tregs that do not produce inflammatory cytokines [33]. In addition, it has also been shown that deletion of Helios contributes to progressive systemic immune activation [34]. We analyzed the proportion of classic CD4^+^CD25^High^FoxP3^+^ Tregs expressing Helios (Fig. 5a) and found an inverse correlation between Tregs expressing Helios and viral load in early infection (*r* = −0.63; *p* = 0.04) (Fig. 5c). This correlation was preserved, when including chronically infected subjects with detectable viral loads (*r* = −0.62; *p* = 0.01) (Fig. 5d). In addition, the proportion of Tregs expressing Helios showed a trend to correlate positively with the recovery of CD4 T cells absolute counts, in early infected group (*r* = 0.6; *p* = 0.056). Furthermore, when adding data from progressive and chronically infected individuals with detectable viral loads, the correlation between Helios expressing Tregs and absolute CD4 T cell counts became significant (*r* = 0.56; *p* = 0.03) (Fig. 5e). We also found tendencies to correlations, between proportions of Tregs expressing Helios in early infected patients with the proportions of activated CD8 T cells (*r* = −0.48; *p* = 0.15) and frequency of antibodies reactive to HIV-1 proteins (*r* = 0.47; *p* = 0.17). The absolute numbers of classic Tregs expressing Helios did not differ significantly between early infected patients with either controls (*p* = 0.24), despite the trend to a decrease of proportions in early infected patients (*p* = 0.06).

## Discussion

Tregs regulate immune responses and prevent or down-regulate exacerbated immune reactions by an array of mechanisms including suppression of T cell activation [35]. This characteristic of Tregs makes them an attractive target to treat inflammatory disorders but also to improve immunogenicity during vaccination against infectious diseases [7, 36, 37]. Tregs are expected to down-regulate immune activation after the acute phase of infection. However, this regulatory activity may also have a negative impact on development of effective anti-HIV immune responses. It has been shown that depletion of regulatory T cells improve reactivation of virus-specific CD8+ T cells in chronic viral infections models such as in Friend retrovirus and in lymphocytic choriomeningitis virus (LCMV) infection [38–40].

During early stages of HIV infection, a massive depletion of CD4 T cells occurs, mainly from the gut associated lymphoid tissue (GALT), the preferential site of initial replication of the virus [41, 42]. Concomitantly, the structure of the GALT is compromised and passive microbial translocation occurs which is believed to drive or contribute to increased non-specific systemic immune activation [42]. We observed an absolute decrease of classic Tregs in patients with HIV early infection, as observed in previous studies [43–45]. However, despite the decrease of the CD4 T cell percentage, we did not find a concordant relative decrease of classic Tregs, suggesting that these cells are better preserved, at least during the early phase of HIV infection. We also found a strong but inverse correlation between Tregs percentages and absolute CD4 counts. Interestingly, high levels of T cell activation were seen in patients with higher percentages of classic Tregs. This may be explained by the fact that classic Tregs are effective in suppressing antigen-specific immune activation but not that of bystander immune activation, as observed in previous HIV studies [46].

Since classic Tregs are more effective in controlling antigen-specific immune responses, we evaluated their association with humoral anti-HIV immune responses. At the early stages of HIV infection, HIV-specific CD8 T cell responses have been shown to be critical, but insufficient to control viral replication [23]. The control of the initial viremia occurs at onset of antibody production to HIV proteins, although it has been suggested that these antibodies are less effective in virus neutralizing and mediating virus inhibition by antibody-dependent cell-mediated cytotoxicity (ADCC) [47]. Here, for the first time, we suggest an involvement of Tregs delaying antibody response in HIV infection. We observed lower frequencies of antibodies reactive to HIV-1 proteins and lower CD4 counts at higher Tregs proportions. In addition, classic Tregs percentages correlated strongly and positively with HIV plasma viral loads. These observations suggest that classic Tregs contribute to the observed delayed onset of antibody production in HIV infection [48], which result in delayed control of viral replication.

The phenotype of Tregs can also predict their organotropism, their target cells, function and susceptibility to HIV infection [27–29, 31]. We assessed the frequency of CXCR3 and CCR5 chemokine receptor expression together with the expression of integrin β7 and the transcription factor Helios. We found a relative increase of Tregs expressing CXCR3 and CCR5 in HIV positive patients with early infection. The proportion of both chemokine receptors was at least twice as high as in uninfected controls. In contrast, the relative and absolute abundance of Tregs expressing the integrin β7 remained unchanged. Despite the higher proportions of CD4 cells expressing the HIV co-receptor CCR5, we did not detect any association between the percentage or absolute counts of CCR5^+^ Tregs on the one hand and the plasma viral load on the other. In previous studies, a higher content of HIV DNA was found in CD25^+^FoxP3^+^ memory CD4 T cells as compared to CD25^−^FoxP3^−^ memory CD4 T cells [30]. However, the sequences of HIV DNA found in memory CD25 + FoxP3+ were not similar to those found in plasma virions [30]. Thus, although Tregs might be preferentially infected by HIV due to expression of the HIV co-receptor CCR5, they probably do not contribute significantly to the overall plasma viral load. Furthermore, it was shown that Tregs are resistant to HIV-1 as consequence of repression of HIV-LTR by FoxP3 [49].

The Helios marker is an ikaros family transcription factor, initially identified as marker of natural Tregs [50, 51]. Helios expression confers stability of phenotype, enhanced immunosuppressive function and increased survival in periphery [34, 52]. Here we show for the first time a correlation between proportions of Helios expressing Tregs with a positive laboratorial outcome in adults with HIV early infection. We found that the frequency of Helios^+^ Tregs remained preserved in patients with early infection, probably due their fitness compared to Tregs not expressing Helios. Interestingly, at higher expression levels of Helios^+^ Tregs, lower viral loads and higher CD4 T cells counts were observed. These results suggest that these bona fide Tregs in addition to being preserved, might be involved in mechanisms controlling the viral replication, probably limiting the activation of T cells. Studies in mice showed that selective deletion of Helios in Tregs leads to slow, progressive systemic immune activation [34]. However, studies conducted in HIV infected children [51] revealed contradictory results regarding correlation of Helios + Tregs with viral loads and absolute CD4 counts. These discrepancies might be explained mainly due to different strategies for identification of Tregs. Since, here we evaluated Helios^+^Tregs as CD4^+^CD25^High^FoxP3^+^Helios^+^ rather than memory Tregs identified as CD4^+^FoxP3^+^Helios^+^CD45RO^+^. In addition, the difference in ages and staging of HIV-1 infection may also contribute to the observed discrepancies.

The limitations of our study are the low sample size, evaluation of only four unique HIV-1 epitopes and the consequent lack of precise staging of the acute infection, based on Fiebig classification and absence of functional tests for assessing Tregs and antibodies functionality.

## Conclusions

Our results suggest that in early HIV infection, classic Tregs do not contribute to the control of systemic T cell activation but rather may be linked to delayed appearance and possibly maturation of antibodies directed against HIV proteins, delaying the control of viral replication. However, highly suppressive Helios expressing Tregs may have a beneficial effect on mechanisms associated with control of viral replication in the periphery. Nevertheless, anti-inflammatory, antiviral and vaccine therapy trials could consider modulation of classic Tregs to evaluate the mechanistic impact of experimental interventions.

## Additional files

Additional file 1: List of antibodies used and a brief description of the purpose. (DOCX 75 kb)

Additional file 2: Gating strategy for identification of CD4 and CD8 T cells and classic Tregs. (PPTX 6278 kb)

Additional file 3: FMO controls for CCR5 and CXCR3 expression, and definition of β7 and Helios on Tregs. (PPTX 6115 kb)

## Abbreviations

AIDS: Acquired Immunodeficiency Syndrome
CISPOC: Centro de Investigação e Treino em Saúde da Polana Caniço
CNBS: Mozambican National Committee on Bioethics in Health (Comité Nacional de Biotética em Saúde de Moçambique)
DMSO: Dimethyl sulfoxide
FCS: Fetal calf serum
Foxp3: (Transcription factor) *forkhead box P3*
HAART: Highly active antiretroviral treatment
HBV: Hepatitis B virus
HCV: Hepatitis C virus
HIV-1: Human immunodeficiency virus type 1
PBMC: Peripheral blood mononuclear cells
Tregs: Regulatory CD4^+^ T cells
