# Supplementary material for: Helios + Regulatory T cell frequencies are correlated with control of viral replication and recovery of absolute CD4 T cells counts in early HIV-1 infection
Source: BMC Immunol. 2017 Dec 16;18:50. doi: 10.1186/s12865-017-0235-7 (PMC5732399; doi:10.1186/s12865-017-0235-7)
Supplement: Supplementary file 1 — List of antibodies used and a brief description of the purpose. (DOCX 75 kb) [file 12865_2017_235_MOESM1_ESM.docx]

**S3.** List of antibodies used and a brief description of the purpose.

| **Marker** | **Clone** | **Fluorochrome** | **Purpose** |
| --- | --- | --- | --- |
| CXCR3 | 1C6/CXCR3 | PerCP | Migration marker. Chemokine receptor |
| CCR5 | 2D7/CCR5 | FITC | Migration marker. Chemokine receptor. HIV co-receptor. |
| 𝛽7 | FIB504 | APC | Integrin. Associated with (α4) integrin, facilitate cellular adhesion and migration to the gut-associated lymphoid tissue. HIV co-receptor. |
| CD31 | WM59 | FITC | Activation status |
| CD45RA | HI100 | PerCP |  |
| HLA-DR | L243 | PE-Cy7 |  |
| CD38 | HIT2 | APC |  |
| FoxP3 | 259D/C7 | PE | Identification of classic regulatory T cells |
| CD25 | M-A251 | PE-Cy7 |  |
| Helios | 22F6 | APC | Subset of classic regulatory T cells |
| HLA-G | 87G | PE | Subset of regulatory T cells |
| CD8 | SK1 | PE-Cy7 | Lineage maker |
| CD3 | SK7 | APC-H7 | Lineage marker |
| CD4 | RPA-T4 | V450 | Lineage marker |
