# Supplementary figures and images for: Helios + Regulatory T cell frequencies are correlated with control of viral replication and recovery of absolute CD4 T cells counts in early HIV-1 infection
Source: BMC Immunol. 2017 Dec 16;18:50. doi: 10.1186/s12865-017-0235-7 (PMC5732399; doi:10.1186/s12865-017-0235-7)

## Slide 1
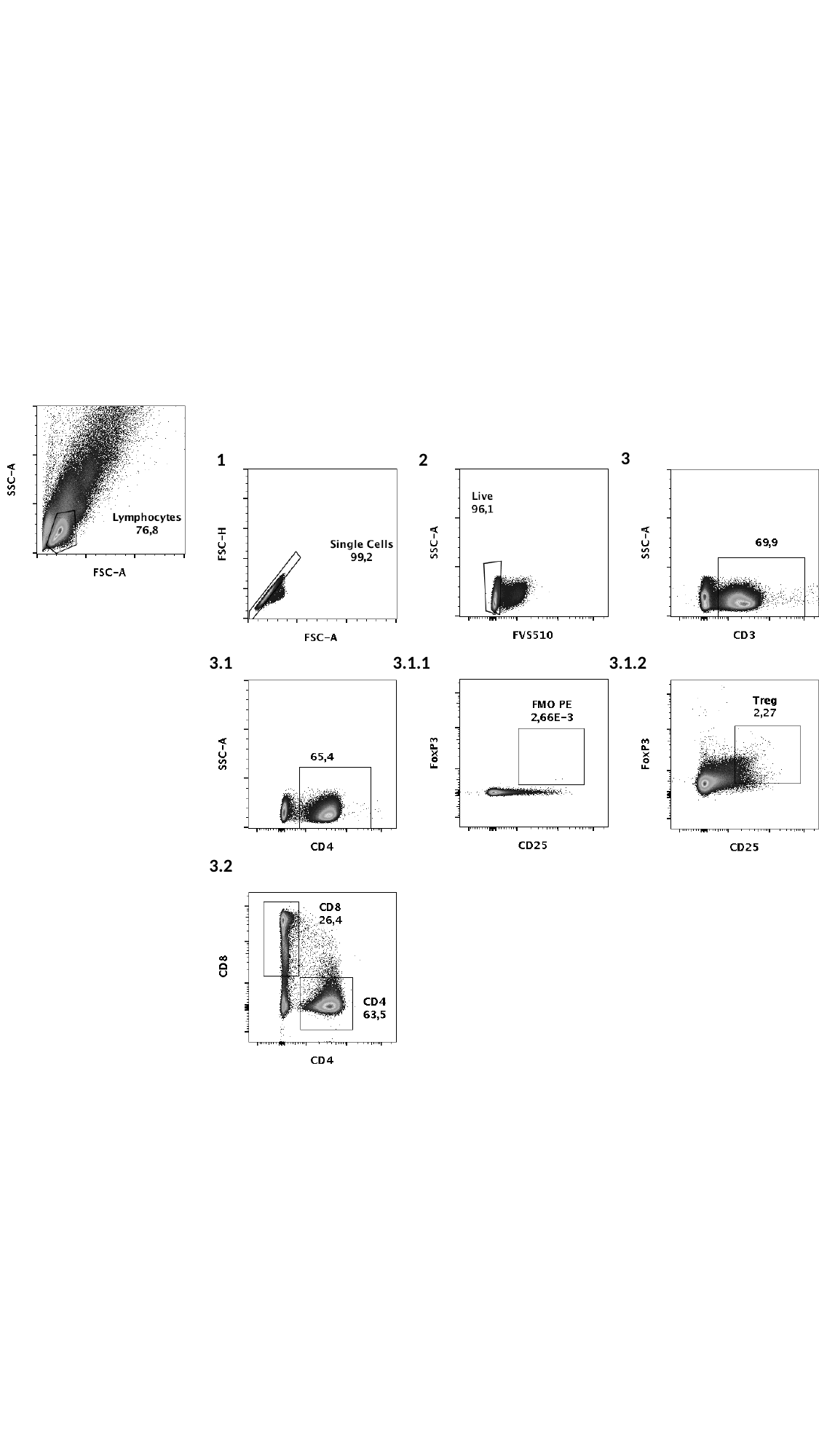

3
1
2
3.1.1
3.1.2
3.1
3.2

Supplement: Supplementary file 2 — Gating strategy for identification of CD4 and CD8 T cells and classic Tregs. (PPTX 6278 kb) [file 12865_2017_235_MOESM2_ESM.pptx]

## Slide 1
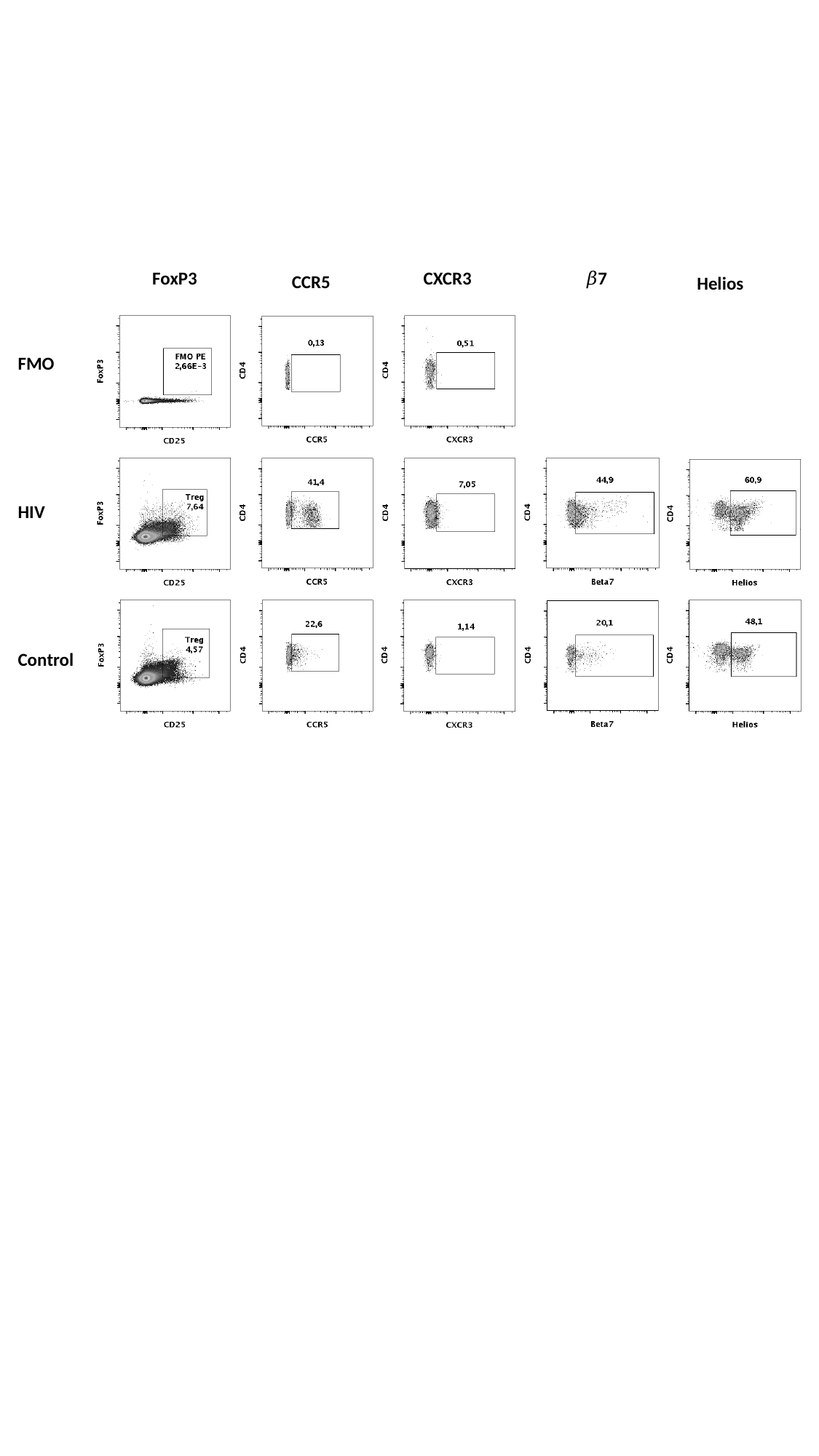

FoxP3
CXCR3
𝛽7
CCR5
Helios
FMO
HIV
Control

Supplement: Supplementary file 3 — FMO controls for CCR5 and CXCR3 expression, and definition of β7 and Helios on Tregs. (PPTX 6115 kb) [file 12865_2017_235_MOESM3_ESM.pptx]
